# Staging Prostate Cancer with ^68^Ga-PSMA-11 PET/CT in the Elderly: Is Preimaging Biopsy Imperative?

**DOI:** 10.2967/jnumed.122.265371

**Published:** 2023-07

**Authors:** Mikhail Kesler, Dan Cohen, Charles Levine, David Sarid, Daniel Keizman, Ofer Yossepowitch, Einat Even-Sapir

**Affiliations:** 1 Department of Nuclear Medicine, Tel Aviv Sourasky Medical Center, Tel Aviv, Israel;; 2 Sackler Faculty of Medicine, Tel Aviv University, Tel Aviv, Israel;; 3 Department of Oncology, Tel-Aviv Sourasky Medical Center, Tel Aviv, Israel; and; 4 Department of Urology, Tel Aviv Sourasky Medical Center, Tel Aviv, Israel

**Keywords:** prostate cancer, staging, PSMA PET/CT, elderly patients, biopsy

## Abstract

Although prostate-specific membrane antigen (PSMA) PET/CT has been shown valuable for staging biopsy-proven [B(+)] high-risk prostate cancer, elderly patients are occasionally referred for PSMA PET/CT without a preimaging confirming biopsy [B(−)]. The current study evaluated the rate, clinical characteristics, and PET-based stage of elderly B(−) patients and explored whether biopsy status affects therapeutic approach. **Methods:** One hundred consecutive patients at least 80 y old who underwent staging ^68^Ga-PSMA-11 PET/CT were included. For each patient, we documented whether preimaging biopsy was performed, the clinical parameters, the PET-based staging parameters, and the primary therapy received. **Results:** Thirty-four (34%) of the elderly patients included in the study had no preimaging biopsy. Compared with B(+) patients, B(−) patients were older (median age, 87 vs. 82 y; *P* < 0.01), with worse performance status (*P* < 0.01) and higher prostate-specific antigen (PSA) levels (median, 57 vs. 15.4 ng/mL; *P* < 0.01). On ^68^Ga-PSMA-11 PET/CT, all B(−) patients had avid disease, with trends toward higher rates of bone metastases (47.1% vs. 28.8%) and overall advanced disease (50% vs. 33.3%) than in B(+) patients. Among patients with localized (*n* = 36) or locally advanced (*n* = 25) disease, B(−) patients were less commonly referred than B(+) patients for definitive therapies (*P* < 0.01). However, higher age, Eastern Cooperative Oncology Group performance status, and PSA were other probable factors determining their therapeutic approach. Among 39 patients with advanced disease, 38 received hormonal therapy irrespective of their biopsy status. Among B(−) patients with advanced disease who were referred for hormonal therapy, 12 of 13 with follow-up data showed a biochemical or imaging-based response. **Conclusion:** Real-life experience with ^68^Ga-PSMA-11 PET/CT indicates that around one third of elderly patients are referred for imaging without a preimaging confirming biopsy. These patients are likely to be older, with a worse clinical status and higher PSA levels. Advanced disease might be more likely to be identified on their ^68^Ga-PSMA-11 PET/CT images, and if it is, their biopsy status does not preclude them from receiving hormonal therapy.

## Visual Abstract

**Figure:**
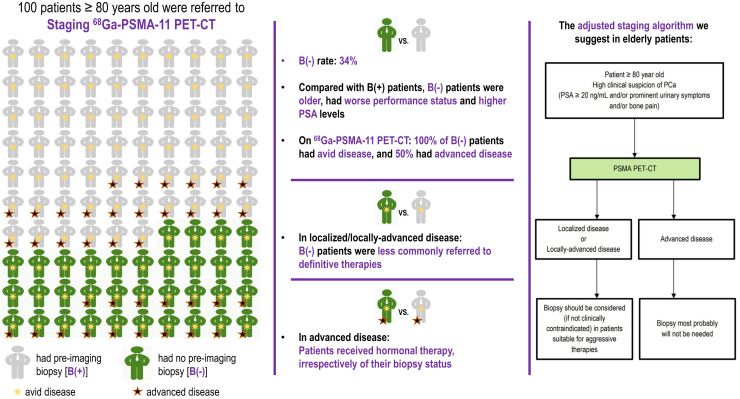

The prostate cancer (PCa) staging algorithm is continually undergoing adjustments. Prostate-specific membrane antigen (PSMA)–based PET imaging is gaining popularity and posing an alternative to traditional imaging modalities (1*–*3). Although first granted a limited approval in the United States in late 2020 (4), PSMA PET/CT was included in the Israeli medical services basket in early 2016 for initial staging of high-risk PCa (5), replacing the traditional use of CT and bone scanning in this group of patients.

On staging, several therapeutic approaches exist for patients with high-risk PCa. Although dictated by guidelines (6*–*8), treatment is generally patient-tailored on the basis of both disease-related and patient-related factors. Patients presenting with high-risk localized cancer are usually considered for definitive therapy by radical prostatectomy or radiotherapy with or without hormonal therapy. Therapy of patients with locally advanced disease usually includes pelvic radiotherapy. Patients with advanced disease are usually offered hormonal therapy aiming to achieve androgen deprivation and delay disease progression. Accurate staging is therefore crucial for optimal treatment planning.

The definition of high-risk PCa is based on clinical T stage (cT3–4), blood prostate-specific antigen (PSA) level (>20 ng/mL), or Gleason score (≥7) (9), and these are the criteria that usually guide clinicians on whether to refer patients for PSMA PET/CT once PCa is histopathologically confirmed. Focusing on elderly patients, however, our impression was that at least some patients are referred for PSMA PET/CT on the basis of high clinical suspicion but without biopsy-proven PCa, probably because of the known inconvenience and potential complications of prostate biopsies (10*,*^11^).

In the current study, we focused on patients at least 80 y old who were referred for PSMA PET/CT with or without a preimaging biopsy confirming the existence of PCa. The aims of the study were to assess the proportion of patients undergoing PSMA PET/CT based on clinical suspicion but without biopsy-proven PCa, identify the characteristics of patients referred on the basis of high clinical suspicion only, calculate the PSMA PET positivity rate in such cases, and explore whether lack of pathologic proof impacted the therapeutic approach selected for these patients.

## MATERIALS AND METHODS

### Patient Population

We retrospectively included all patients who met the following criteria: had an age of at least 80 y, underwent whole-body ^68^Ga-PSMA-11 PET/CT in the department of nuclear medicine at Tel-Aviv Sourasky Medical Center for primary staging between January 2016 and September 2021, and were clinically evaluated and treated at Tel-Aviv Sourasky Medical Center. A total of 100 patients fulfilled the study’s inclusion criteria. The study protocol was approved by the local institutional ethics committee, which waived the requirement for written informed consent (approval TLV-0327-20).

#### Preimaging Clinical Data

For all included patients, we documented the following clinical parameters: age, PSA level, Eastern Cooperative Oncology Group performance status, and whether a preimaging biopsy was performed, B(+), or not performed, B(−). If the patient underwent a biopsy, we documented the highest International Society of Urological Pathology group grade reported on histopathologic assessment.

#### PET/CT-Based Staging Data

For all included studies, we documented the disease extent identified on PSMA PET/CT imaging using the European Association of Nuclear Medicine standardized reporting guidelines for PSMA PET (12): miT stage, that is, evidence of a local avid tumor (organ-confined, suspected extracapsular extension, seminal vesicle invasion, invasion to other adjacent structures); miN stage, that is, evidence of regional avid nodal disease (pelvic nodes); and miM stage, that is, evidence of avid distant metastases (extrapelvic nodes, bone or visceral metastases). Accordingly, patients’ malignancies were categorized into 3 groups: localized disease (malignancy that involves the prostate only), locally advanced disease (extraprostatic extension or regional nodal disease, without distant metastases), and advanced disease (evident distant metastases) (13*–*15). For each case, the PET/CT-based staging parameters were recorded in the dataset on the basis of the data interpretation and categorization that appeared in the final PET/CT report. Beyond the PET/CT imaging data, all available data regarding the patient’s previous clinical history and course of the disease, and all available data from previous laboratory, pathologic, and imaging studies, were used to assist in imaging interpretation and categorization.

#### Therapy Data

For each included patient, we documented which of the following therapies were used after staging PET/CT: radical prostatectomy, pelvic radiotherapy, hormonal therapy (androgen deprivation therapy or other hormonal therapies), or other systemic therapies. If none of these were given, the patient was considered to have been put under surveillance (a watch-and-wait strategy).

### PET/CT Imaging

PET/CT studies were performed from the tip of the skull to mid thigh using Discovery 690 or Discovery MI PET/CT systems (GE Healthcare). An activity of 148–166 MBq of ^68^Ga-PSMA-11 was intravenously injected 60 min before acquisition. The patients were instructed to void their bladder immediately before the acquisition. Contrast material was administered orally and intravenously, unless contraindicated. CT was performed using automatic mA-modulation and 120 kV. CT scans were reconstructed to a slice thickness of 2.5 mm. PET was performed with an acquisition time of 2.5–3 min per bed position in 3-dimensional mode. PET images were reconstructed in a matrix size of 128 × 128, pixel size of 5.5 mm, and slice thickness of 3.3 mm for the Discovery 690 system and a matrix size of 256 × 256, pixel size of 2.7 mm, and slice thickness of 2.8 mm for the Discovery MI system. The reconstruction method was VUE Point FX (GE Healthcare), which uses time-of-flight information and includes a fully 3-dimensional ordered-subsets expectation maximization algorithm with 3 iterations. Reconstruction used 24 subsets and a filter cutoff of 8 mm for the Discovery 690 system and 8 subsets and a filter cutoff of 6 mm for the Discovery MI system. The VUE Point FX algorithm also includes normalization and image corrections for attenuation, scatter, randoms, and dead time. A heavy Z-filter was applied to smooth between transaxial slices.

### Statistical Analysis

Categoric data were described with contingency tables that included frequency and percentage. Continuous variables were evaluated for normal distribution and reported as median and interquartile range. The Pearson χ^2^ test and Fisher exact test were used to compare rates of categoric variables. The Mann–Whitney *U* test was used to compare medians of continuous variables between 2 unpaired groups. A 2-sided *P* value of less than 0.05 was considered statistically significant. SPSS software (IBM SPSS Statistics, version 27; IBM Corp.) for Microsoft Windows was used for statistical analysis.

## RESULTS

### Patient Characteristics

The median patient age was 82 y (interquartile range, 80–86.8 y; range, 80–94 y). At the time of imaging, 33% and 11% of patients were at least 85 y old and at least 90 y old, respectively. The median blood PSA level was 22.6 ng/mL, with 53% and 13% of patients having a PSA level above 20 and 100 ng/mL, respectively.

From their ^68^Ga-PSMA-11 PET/CT studies, 36 and 25 patients were categorized as having localized and locally advanced disease, respectively. The other 39 patients had advanced disease: 35 had bone metastases, 10 had distal nodal disease, 3 had liver metastases, 2 had lung metastases, and 1 had peritoneal involvement. Table 1 summarizes the clinical, staging, and therapy data of the total study patients.

**TABLE 1.** Patient Characteristics (*n* = 100)

Variable	Value
Preimaging clinical data	
Age (y)	82 (80–86.8)
ECOG PS = 0	52 (52%)
ECOG PS = 1	39 (39%)
ECOG PS ≥ 2	9 (9%)
PSA (ng/mL)	22.6 (9.4–59.0)
PSA < 5	6 (6%)
5 ≤ PSA < 10	22 (22%)
10 ≤ PSA < 20	19 (19%)
PSA > 20	53 (53%)
PSMA PET–based staging data	
Avid disease	100 (100%)
Localized disease	36 (36%)
Locally advanced disease	25 (25%)
Advanced disease	39 (39%)
Therapy data	
Radical prostatectomy	2 (2%)
Radiotherapy	36 (36%)
Hormonal therapy	79 (77%)
Surveillance	14 (14%)

ECOG PS = Eastern Cooperative Oncology Group performance status.

Categoric variables are reported as frequency and percentage. Continuous variables are reported as median and interquartile range.

### Characteristics of Patients Undergoing PSMA PET/CT Without Preimaging Biopsy

Of the 100 patients, 34 (34%) were referred for ^68^Ga-PSMA-11 PET/CT on the basis of high clinical suspicion only, without preimaging histopathologic confirmation of PCa (Table 2. Eighteen (53%) of these 34 were referred after documentation of significantly elevated PSA levels (>50 ng/mL), 15 (44.1%) were referred because of prominent clinical symptoms (11 had urinary symptoms, 4 had bone pain), and 1 (2.8%) was referred because of focal prostatic ^18^F-FDG uptake observed on ^18^F-FDG PET/CT performed for colon cancer follow-up. Among these 34 B(−) patients, 10 (29.4%) underwent prostate MRI before referral for PSMA PET/CT, with the highest reported Prostate Imaging–Reporting and Data System score (4 or 5) documented in 9 of them.

**TABLE 2.** Comparison Between B(+) and B(−) Patients

Variable	B(+) (*n* = 66)	B(−) (*n* = 34)	*P*
Preimaging clinical data			
Age (y)	82 (80–83)	87 (83–90)	<0.01*
ECOG PS = 0	43 (65.2%)	9 (26.5%)	<0.01*
ECOG PS = 1	22 (33.3%)	17 (50%)	0.10
ECOG PS ≥ 2	1 (1.5%)	8 (23.5%)	<0.01*
PSA (ng/mL)	15.4 (8.3–43.2)	57 (22.4–82.5)	<0.01*
PSA < 5	6 (9.1%)	0 (0%)	0.09
5 ≤ PSA < 10	19 (28.8%)	3 (8.8%)	0.02*
10 ≤ PSA < 20	15 (22.7%)	4 (11.8%)	0.19
PSA > 20	26 (39.4%)	27 (79.4%)	<0.01*
ISUP GG 1	4 (6.1%)	—	—
ISUP GG 2	16 (24.2%)	—	—
ISUP GG 3	14 (21.2%)	—	—
ISUP GG 4	10 (15.2%)	—	—
ISUP GG 5	22 (33.3%)	—	—
PSMA PET-based staging data			
Avid disease	66 (100%)	34 (100%)	—
Localized disease	27 (40.9%)	9 (26.5%)	0.15
Locally advanced disease	17 (25.8%)	8 (23.5%)	0.81
Advanced disease	22 (33.3%)	17 (50%)	0.11
Therapy data			
Radical prostatectomy	2 (3%)	0 (0%)	0.55
Pelvic radiotherapy	35 (53%)	1 (2.9%)	<0.01*
Hormonal therapy	54 (81.8%)	25 (73.5%)	0.34
Surveillance	5 (7.6%)	9 (26.5%)	0.02*

* Statistically significant.

ECOG PS = Eastern Cooperative Oncology Group performance status; ISUP GG = International Society of Urological Pathology grade group.

Categoric variables are reported as frequency and percentage. Continuous variables are reported as median and interquartile range.

Compared with B(+) patients (Table 2), B(−) patients were older (median age, 87 vs. 82 y; *P* < 0.01) and had worse performance status scores (Eastern Cooperative Oncology Group performance status ≥ 2 in 23.5% vs. 1.5%, *P* < 0.01). The PSA level of these patients was significantly higher (median, 57 vs. 15.4 ng/mL; *P* < 0.01), with 79.4% and 17.7% of them having PSA levels higher than 20 and 100 ng/mL, respectively.

**FIGURE 1.**
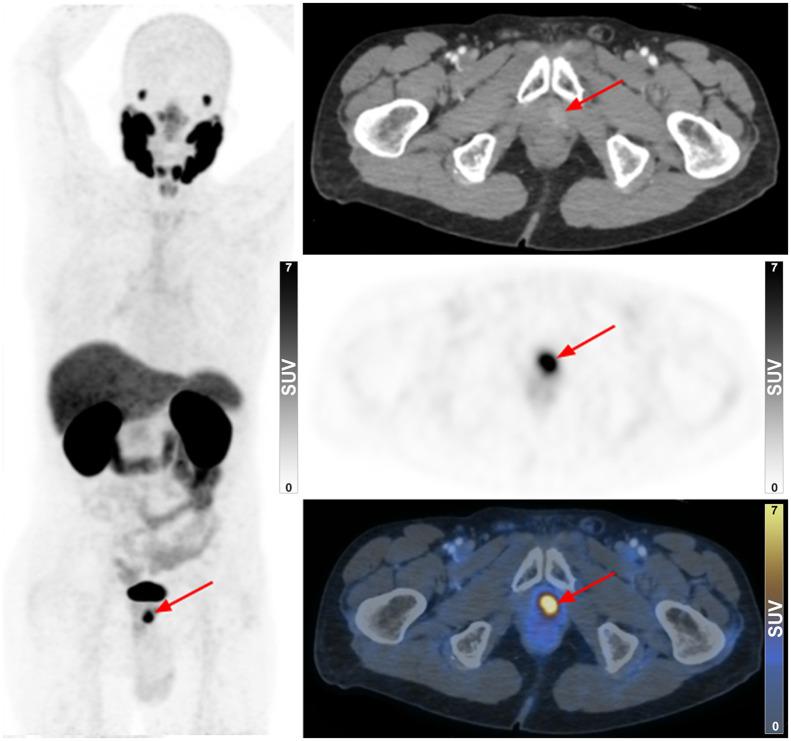
^68^Ga-PSMA-11 PET/CT of 84-y-old B(−) man with rising PSA levels. Focus of increased uptake was identified at left apex of prostate (arrow), indicating localized disease.

**FIGURE 2.**
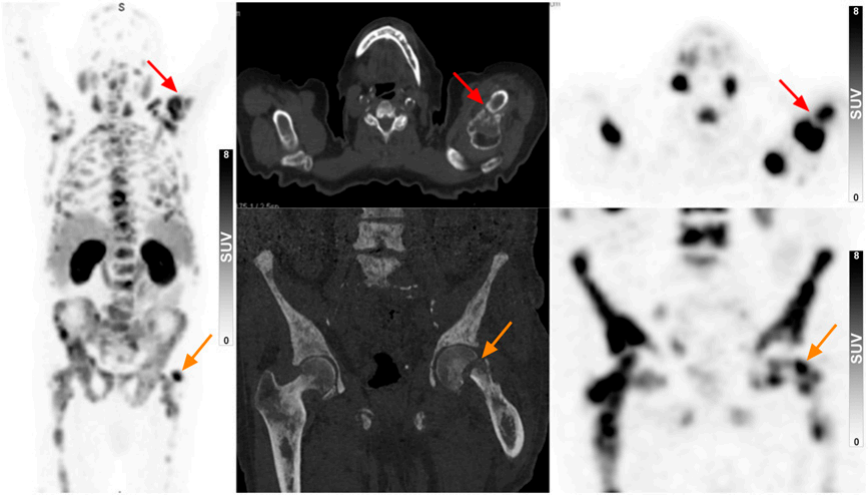
^68^Ga-PSMA-11 PET/CT of 82-y-old B(−) man with bone pain and PSA levels of 960 ng/mL. Not only effective in identifying disease extent, study also detected pathologic fractures of left humerus and left femur (arrows). Patient was immediately referred for radiotherapy for humeral lesion and surgery for femoral lesion.

In B(−) patients and B(+) patients, 100% of them (34/34 and 66/66, respectively) had at least 1 avid lesion that was considered to represent PCa on their ^68^Ga-PSMA-11 PET/CT scan. Examples of a B(−) patient with localized disease and a B(−) patient with findings that required special clinical attention are presented in Figures 1 and 2, respectively. In terms of staging parameters, the B(−) patients had no staging parameters significantly different from the B(+) patients, although trends toward higher rates of bone metastasis (47.1% vs. 28.8%) and overall advanced disease (50% vs. 33.3%) were noted in these patients.

### Is Preimaging Biopsy a Factor in Choosing Therapeutic Approach?

Comparing therapy approaches among the total study cohort (Table 2), similar proportions of B(+) and B(−) elderly patients were referred for hormonal therapy (81.8% and 73.5%, respectively, *P* = 0.34). In contrast, whereas 63% of B(+) patients were referred for radiotherapy after imaging, only 8.8% of B(−) were referred for radiotherapy (*P* < 0.01, Table 2). To better assess the differences in therapeutic approach selected for B(−) versus B(+) patients, therapies given to patients in 3 different stage groups were analyzed: localized disease, locally advanced disease, and advanced disease (Table 3).

**TABLE 3.** Factors Affecting Therapeutic Approach, Stratified by Disease Extent

Parameter	Therapeutic approach 1	Therapeutic approach 2	*P*
Localized disease (*n* = 36)			
Therapy type	Definitive therapy* (*n* = 22)	Other therapy (*n* = 14)	
B(+) patients (*n* = 27)	22	5	<0.01^†^
B(−) patients (*n* = 9)	0	9	<0.01^†^
Age (y)	82 (80–83)	82.5 (81.8–86.5)	0.10
ECOG PS ≥ 1	5/22 (22.7%)	9/14 (64.3%)	0.01^†^
PSA (ng/mL)	10.4 (7.2–18.2)	14.8 (8.4–22.3)	0.31
Locally advanced disease (*n* = 25)			
Therapy type	Pelvic radiotherapy (*n* = 16)	Other therapy (*n* = 9)	
B(+) patients (*n* = 17)	15	2	<0.01^†^
B(−) patients (*n* = 8)	1	7	<0.01^†^
Age (y)	80 (80–81.8)	87 (85.5–92)	<0.01^†^
ECOG PS ≥ 1	6/16 (37.5%)	6/9 (66.7%)	0.23
PSA (ng/mL)	13.5 (6.7–29.5)	56 (28.4–74)	<0.01^†^
Extraprostatic extension	8/16 (50%)	6/9 (66.6%)	0.68
Regional nodal disease	11/16 (68.8%)	8/9 (88.9%)	0.36
Advanced disease (*n* = 39)			
Therapy type	Hormonal therapy (*n* = 38)	Other therapy (*n* = 1)	
B(+) patients (*n* = 22)	22	0	0.44
B(−) patients (*n* = 17)	16	1	0.44

* Radical prostatectomy or pelvic radiotherapy.

† Statistically significant.

ECOG PS = Eastern Cooperative Oncology Group performance status.

Categoric variables are reported as frequency and percentage. Continuous variables are reported as median and interquartile range.

#### Localized Disease

Among the 36 patients with localized disease, 22 were referred for definitive therapy (20 patients underwent pelvic radiotherapy and 2 patients underwent radical prostatectomy), and 14 patients were given nondefinitive therapy (4 received hormonal therapy, and 10 were put under surveillance). B(−) patients were less commonly referred for definitive therapies than B(+) patients (0/9 vs. 22/27, 0% vs. 81.5%, *P* < 0.01). Still, beyond biopsy status, those referred for definitive therapy also had a statistically significant better performance status (*P* = 0.01) and a trend toward a younger median age (*P* = 0.10).

#### Locally Advanced Disease

Among the 25 patients with locally advanced disease, 16 were referred for pelvic radiotherapy (14 received hormonal therapy as well). The other 9 received hormonal therapy only (*n* = 6) or were put under surveillance only (*n* = 3). Similarly to patients with localized disease, among patients with locally advanced disease, B(−) patients received pelvic radiotherapy at significantly lower rates than B(+) patients (1/8 vs. 15/17, 12.5% vs. 88.2%, *P* < 0.01). However, age and PSA level were significantly lower in those receiving radiotherapy (*P* < 0.01 for both variables).

#### Advanced Disease

Among the 39 patients with advanced disease, 38 received hormonal therapy (9 were referred for radiotherapy as well) and 1 was put under surveillance. In contrast to patients with localized or locally advanced disease, B(−) and B(+) patients with advanced disease received hormonal therapy at similar rates (16/17 and 22/22, 94.1% and 100%, *P* = 0.44), indicating that biopsy was not a factor determining the therapy approach in these patients.

**FIGURE 3.**
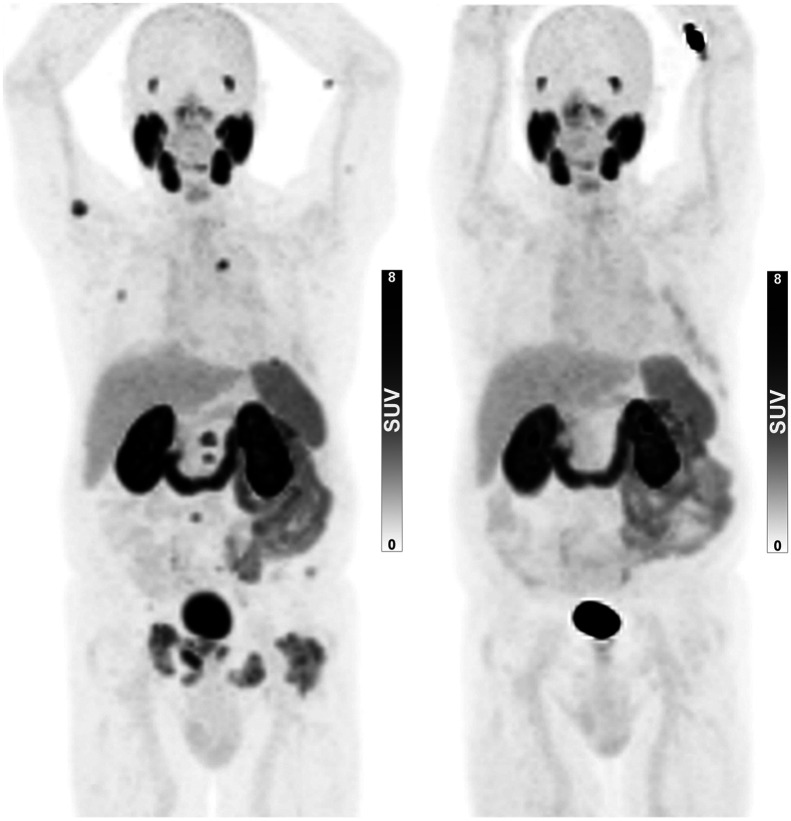
^68^Ga-PSMA-11 PET/CT scans of 83-y-old B(−) man before (left) and after (right) initiation of hormonal therapy. Staging scan (left) was performed without preimaging biopsy, revealing advanced disease. On imaging performed 10 mo later (right), all previous PSMA-positive lesions demonstrated no uptake of ^68^Ga-PSMA-11. PSA level of this patient decreased dramatically on therapy initiation as well (from 67 to 3.1 ng/mL).

Focusing on the 16 B(−) patients whose PSMA PET/CT revealed advanced disease and were then put on hormonal therapy (with no preimaging or pretherapy confirming biopsy), follow-up data on their next PSA level and next PSMA PET/CT were available for 13 and 9 patients, respectively. In 12 (92.3%) of the 13 patients with documented PSA levels, a dramatic decrease in PSA levels was found (mean change, 95.6%; interquartile range, 95.0%–98.2%). In 8 (88.9%) of the 9 patients with repeated PSMA PET/CT, either a significant decrease in PSMA-avid disease extent (*n* = 5, Fig. 3) or stable disease (*n* = 3) was documented. Only 1 patient was found to have increasing PSA levels and progressive disease on imaging after hormonal therapy initiation.

## DISCUSSION

Although ^68^Ga-PSMA-11 PET/CT is indicated primarily for staging of patients with biopsy-proven high-risk PCa, real-life experience indicates that elderly patients are occasionally referred for imaging on the basis of high clinical suspicion alone without prior histopathologic confirmation of PCa. This single-center retrospective study explored this practice among elderly patients and found it to occur in around one third of patients at least 80 y old.

Previous literature indicates that in elderly patients, prostate biopsies are associated with longer hospital stays (10), and postbiopsy complications such as infection, bleeding, or urinary retention might occur in of up to 17% of cases (11). Moreover, elderly patients are prone to delayed clinical management and reduced compliance with invasive procedures (16). Therefore, reduction of unnecessary biopsies and effective diagnosis of clinically significant PCa in elderly patients should be a medical priority. Previous studies that contain data on the application of PSMA PET/CT before biopsy were not directed to the population of elderly patients and did not focus on the potential of imaging to eliminate the need for biopsy in the elderly (or in other predefined populations). Instead, these studies evaluated mainly the diagnostic performance and the prognostic information provided by PSMA PET/CT, using postimaging histopathologic data as a reference (17*,*^18^). The results of the current study indicate that 100% of the elderly patients who were referred for PSMA PET/CT on the basis of clinical suspicion only (mainly a rising PSA level or prominent urinary or musculoskeletal symptoms) were found to have avid disease on PSMA PET/CT. Given that positive PSMA PET/CT results usually indicate clinically significant rather than clinically insignificant PCa (19), and together with insignificant negative biologic effect of radiation exposure in elderly patients (20), this practice of waiving the need for preimaging biopsy when the clinical suspicion is high proves to be effective and to have no apparent negative cost. This practice is not common in the field of PET/CT but has been shown cost-effective and adopted in the assessment algorithm of solitary pulmonary nodules (21).

By characterizing the patients undergoing PSMA PET/CT without preimaging biopsy, whose PET-positivity rate was 100%, the current study provides practical tools for clinicians. It seems that PSMA PET/CT is highly likely to identify and stage PCa in patients at least 80 y old with a PSA level of at least 20 ng/mL, even without histopathologic confirmation.

As for postimaging therapy, our results indicate that preimaging biopsy may play a role in selecting the therapeutic approach toward localized or locally advanced PCa cases. Those patients in our cohort who did not undergo preimaging biopsy were less likely to get definitive therapies if having localized or locally advanced disease. However, it seems that those not referred for definitive therapies were usually older, with worse performance status or higher PSA levels. These data support the probability that biopsy status was not the sole factor on which the therapy approach was based and that other patient-related factors probably affected therapy selection as well. These observations suggest that a selected group of patients with localized or locally advanced PCa, who are candidates for more aggressive therapies after PSMA PET/CT, should be considered for a complete postimaging histopathologic assessment.

When PSMA PET/CT identified advanced disease in patients who had no preimaging biopsy, as happened in half of such patients in the current study, our results indicate that the lack of tissue diagnosis did not preclude them from receiving hormonal therapy, which biochemical and imaging follow-up data indicated had been beneficial.

**FIGURE 4.**
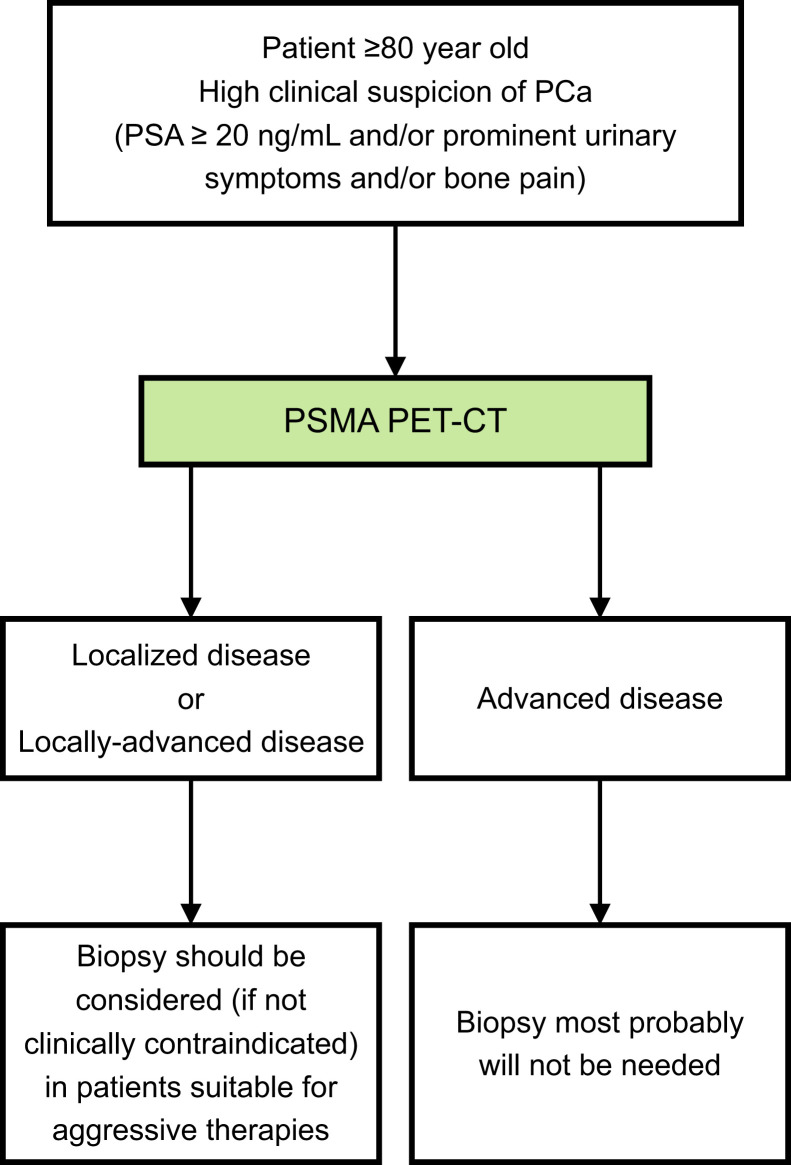
Adjusted staging algorithm for elderly patients for whom there is high clinical suspicion of PCa.

On the basis of our results, we outlined in Figure 4 an adjusted PCa staging algorithm for elderly patients. Adoption of this suggested algorithm can potentially reduce the numbers of prostate biopsies and associated complications in the elderly while providing accurate staging data. If patients are selected correctly for imaging, the risk of performing unnecessary PSMA PET/CT seems negligible. Still, larger studies are required to validate our results. If confirmed in additional series, our proposed algorithm could be implemented in the PCa staging algorithm for elderly patients.

## DISCLOSURE

No potential conflict of interest relevant to this article was reported.

## Notes

KEY POINTS **QUESTION:** How commonly are elderly patients referred for staging PSMA PET/CT without preimaging biopsy, what is the PET-positivity rate in such patients, are their clinical and PET-based staging parameters different from those of patients referred with biopsy, and does biopsy status affect the selected therapeutic approach? **PERTINENT FINDINGS:** In real life, around one third of elderly patients are referred for PSMA PET/CT without a preimaging confirming biopsy. These patients are likely to be older, with worse clinical status and a higher PSA level, and avid disease is expected to be identified on their PSMA PET/CT images. Identification of advanced disease may be more likely in these patients, and if it is, their biopsy status does not preclude them from receiving hormonal therapy, which results in a biochemical and imaging-based response. **IMPLICATIONS FOR PATIENT CARE:** In elderly patients with a high clinical suspicion of PCa, an excellent PET-positivity rate is provided by PSMA PET/CT, even without preimaging biopsy. Advanced disease identified on PSMA PET/CT can be considered confirmatory of PCa and serve as a criterion to initiate hormonal therapy, potentially waiving the need for preimaging biopsy in frail patients.
